# Out of fright, out of mind: impaired memory for information negated during looming threat

**DOI:** 10.1186/s41235-021-00302-4

**Published:** 2021-05-07

**Authors:** Vera E. Newman, Hannah F. Yee, Adrian R. Walker, Metaxia Toumbelekis, Steven B. Most

**Affiliations:** School of Psychology, UNSW Sydney, Sydney, NSW 2052 Australia

**Keywords:** Memory updating, Memory re-evaluation, Memory interference, Misinformation, Anxiety

## Abstract

People often need to update representations of information upon discovering them to be incorrect, a process that can be interrupted by competing cognitive demands. Because anxiety and stress can impair cognitive performance, we tested whether looming threat can similarly interfere with the process of updating representations of a statement’s truthfulness. On each trial, participants saw a face paired with a personality descriptor. Each pairing was followed by a signal indicating whether the pairing was “true”, or “false” (a negation of the truth of the statement), and this signal could be followed by a warning of imminent electric shock (i.e., the looming threat). As predicted, threat of shock left memory for “true” pairings intact, while impairing people’s ability to label negated pairings as untrue. Contrary to our predictions, the pattern of errors for pairings that were negated under threat suggested that these mistakes were at least partly attributable to participants forgetting that they saw the negated information at all (rather than being driven by miscategorization of the pairings as true). Consistent with this, linear ballistic accumulator modelling suggested that this impaired recognition stemmed from weaker memory traces rather than decisional processes. We suggest that arousal due to looming threat may interfere with executive processes important for resolving competition between mutually suppressive tags of whether representations in memory are “true” or “false”.

## Statement of relevance

The rapid pace of government- and media-information output almost guarantees that the public will often encounter initially incorrect information that subsequently needs to be corrected, negated, or retracted. The importance of updating one’s representation of information’s veracity is heightened when the information involves advice for responding to anxiety provoking situations (e.g., an emerging pandemic or wildfires). Thus, the current findings that people have impaired memory for information that is negated during potential threat provide insight into pitfalls that must be avoided when agencies convey information about anxiety-provoking events. The current findings provide evidence and a potential mechanism for why it can sometimes be more important to slow the output and verify the accuracy of important information than to quickly convey it and rely on corrective negations of a statement.

Woe to you if you were low on toilet paper in the early days of COVID-19. Early media reports of panic buying in preparation for extended periods of self-quarantine led supermarket shelves to be emptied of such products within days. After a short delay, experts weighed in to suggest that this strategy was unproductive, that such hoarding was unnecessary, and that there were better ways to prepare for the looming pandemic, but this led only to a slow course correction. The shelves remained bare, and those who arrived late to the store were caught feeling a little behind.

Given the scope and seriousness of the COVID-19 crisis, #toiletpapergate is destined to be a footnote to one of the most disruptive events in modern history. But it serves as an important lesson: in emergencies, the public needs access to information and guidance, and the fast pace of information output means that much of what they initially encounter will be not accurate. Thus, it is important that people are able to reclassify information as false in order to keep track of what advice to prioritize and what to discard. Despite the importance of doing so, the influence of misinformation has been found to persist even after it has been rescinded (i.e., “negated”; Ecker et al. 2011; Johnson and Seifert 1994; Wilkes and Leatherbarrow 1988). Ironically, situations that are anxiety-provoking may be ones that simultaneously lead to both a flood of initially incorrect information and an impaired ability to apply corrections when misleading information is identified. The current study aimed to assess the second of these possibilities: that anxious anticipation of an aversive outcome might selectively impair how people process information that they have been told is not true.

This possibility would be consistent with findings suggesting that people initially accept information as true and take corrective information into account only if they have the cognitive resources to do so (Gilbert et al. 1990; Wilson and Park 2008). In one study, participants learned a series of ostensibly Hopi words (e.g., “A *tica* is a fox”), and each word-translation pair was followed by the signal word “True” or “False”, which indicated whether participants should regard the translation as accurate or not (Gilbert et al. 1990). On a subset of trials, the signal word was immediately followed by a tone that required a manual response from the participant. This secondary task served to interrupt the process of “unaccepting” negated translations. When participants were later tested on their ability to recall the veracity of the translations, they were more likely to misremember false translations as true if the “False” signal word had immediately been interrupted by a tone. In contrast, accuracy was not impaired when the interruption followed the “True” signal word. In their paper, Gilbert and colleagues thus hypothesized that people initially accept information that they receive as truthful, and then “unaccept” that information if necessary.

Findings that cognitive interruptions can impair people’s ability to apply such negations, combined with findings that anxiety and stress can interfere with cognitive processing (Vytal et al. 2012; Vytal et al. 2012), highlight the possibility that anxiety or stress on their own can make it more difficult for people to mentally update their representations of the veracity of information. If so, then there are implications for how information is presented for public consumption. Today’s 24-h news cycle relentlessly broadcasts highly charged, anxiety-provoking information at a rate that almost guarantees a need to correct or retract some of it (Slone 2000; McNaughton-Cassill 2001).

In the current study, we aimed to examine the impact of looming threat, operationalized by threat of shock, on people’s ability to reclassify information as false following a negation. Roughly following the procedure reported by Gilbert and colleagues (1990), participants learned pairings between stimuli—in this case, they saw faces, each of which was followed by a word describing a trait associated with that face. Based on Gilbert and colleagues’ hypothesis, these face-descriptor pairs should all be initially classified as truthful by the receiver. However, each of these face-word pairs was followed by the signal word “True” or “False” to indicate whether participants should continue to regard the word as an accurate description of the person, or whether they should reclassify their mental representation of the pairings as being false in light of the negatory information. Instead of a tone, each signal word was followed by a symbol indicating whether or not participants were likely to receive an electric shock. This manipulation allowed us to test whether anticipation of an aversive stimulus selectively impaired participants’ ability to recognize that information had been negated. We hypothesized that anxious anticipation, as operationalized by threat of shock, would selectively impair people’s ability to apply a negation of false information, resulting in reduced accuracy in identifying face-descriptor pairings that were negated (i.e., those indicated as false), but intact accuracy in identifying pairings that were not negated (i.e., those that were indicated to be true). We hypothesized that this pattern would not emerge when true/false signal words appeared in the absence threat of shock.

Additionally, the descriptors associated with the faces were selected to be positive, negative, or neutral traits. This aspect of the design explored whether any impact of threat anticipation on memory for negated or non-negated information was modulated by the information’s emotional valence.

## Method

### Participants

One hundred and twenty-one women (Mean Age_years_  =  20.11, SD  =  4.25) were recruited from the University of New South Wales (UNSW Sydney) in return for course credit or monetary payment. Participants were excluded from participating if they were not native English speakers or reported a current or previous heart condition. Males were not recruited to avoid potential sex-specific differences in visual memory for male and female faces, previously found in literature (Lewin and Herlitz 2002; Wright and Sladden 2003). The study was approved by UNSW’s Human Research Ethics Advisory Panel.

### Measures

#### Learning phase

**Fig. 1:**
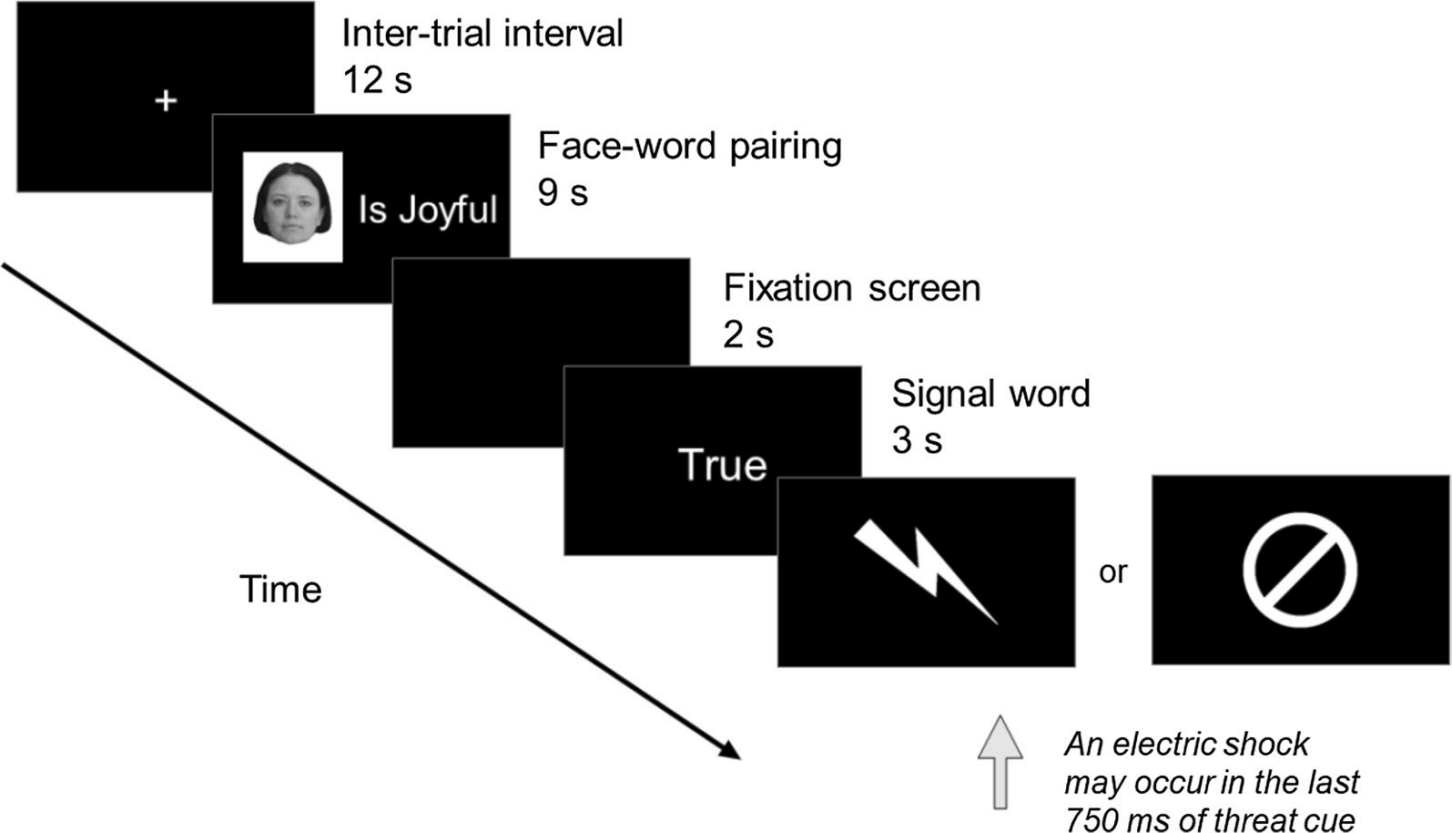
Sequential representation of events from a learning trial. At the end of each learning phase trial, participants were shown either a threat cue indicating possible shock (threat trial) or a safety cue (safe trial). On a subset of the threat trials, the cue actually was followed by an electric shock (shock trial)

Participants completed 15 learning trials in each of four experimental blocks. On each trial in the learning blocks, participants saw a face alongside a word descriptor for 9 s, followed by a 2 s fixation screen. Following the fixation, a signal word “TRUE” or “FALSE” was shown for 3 s to indicate the veracity of the face-descriptor pairing (i.e., whether the word accurately described the personality of the paired face). This was followed by a 1 s cue indicating whether or not there was the potential of imminently receiving electric shock (see Fig. 1). Inter-trial intervals of 12 s were incorporated to allow physiological response to the shock to return to baseline.

Each block contained six *safe* trials (i.e., a trial on which a cue signalling safety was shown), six *threat* trials (threat cue), and three *shock* trials (threat cue + shock delivery). While the number of trials of a specific type was fixed within blocks, the stimuli presented for each of these trial types, and the order of trials within blocks, was randomized across participants. On shock trials, 500 ms of electrical stimulation was delivered to the participant’s forearm via a stimulus isolator (Model FE180, ADInstruments), 750 ms before the offset of the cue (pulse width  =  1 ms, frequency  =  500 Hz, voltage  =  5 V; current was adjusted for each participant as described below). On half of each of the safe and threat trials per block, the word “TRUE” followed the pairing, and the word “FALSE” followed the remaining half. Because each block contained an odd number (3) of actual shock trials, the distribution of instances where the signal word “TRUE” or “FALSE” was followed by shock was balanced across the first pair and the second pair of experimental blocks.

To help ensure that task performance was not driven by an inability to differentiate faces from each other, the face stimuli were 84 female faces previously rated as visually dissimilar from each other, taken from the Glasgow Unfamiliar Face Database (Burton et al. 2010). Word descriptors were 28 positive, 28 neutral, and 28 negative emotional words from the Warriner, Kuperman, and Brysbaert’s dataset of affective norms (2013; see Additional file 1 for further details about the face and word stimuli). Faces and descriptors were randomly paired to create a face-descriptor pairing, with each block employing five positive, five neutral, and five negatively valenced words. Shock trials were counterbalanced across pairs of blocks, where two negative, two neutral, and two positive descriptors were pseudo-randomly chosen for the six shock trials within the two blocks.

#### Memory phase

**Fig. 2:**
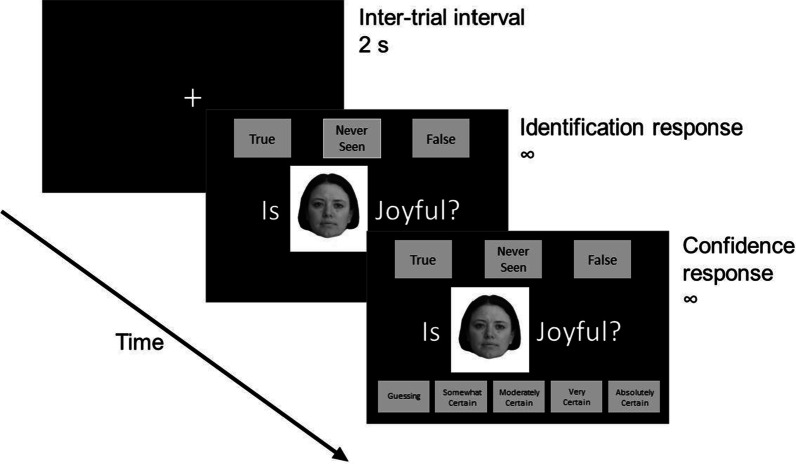
Sequential representation of events from a memory trial. At the end of each learning phase, participants were shown previously seen and novel face-descriptor pairings and were asked to indicate whether they were true, false, or never seen before. Participants were also asked to rate their confidence for each judgement

Following each learning block, participants were tested on their memory for that block’s face-word pairings and their veracity (see Fig. 2). On each trial, participants saw, in random order, a face-descriptor pairing from the prior learning phase or a new face-descriptor pairing (foils), and they were asked whether the pairing was “True”, “False,” or “Never Seen” (i.e., if they had not seen the face-descriptor during the learning phase). Response times were recorded, and after each response participants rated their confidence in their response on a 1–5 scale, “Guessing”, “Somewhat Certain”, “Moderately Certain”, “Very Certain”, or “Absolutely Certain”. Each memory phase included a randomly ordered presentation of the 15 face-word pairings from the prior learning phase, and six foil trials of novel face-descriptor pairings. Of these foil trials, a novel face was randomly paired with a novel descriptor, with random assignment of two positive, two negative, and two neutral descriptors.

#### Physiological and affective responses

Throughout all experimental blocks, participants’ skin conductance response (SCR) was measured via a finger electrode on their middle and index fingers as an index of physiological arousal (Bach 2014). Shock delivery and SCR recordings were controlled by MATLAB (Brainard 1997; Pelli 1997) and LabChart 8 software (ADInstruments). Participants also completed a self-report questionnaire to index their overall arousal, subjective feelings of stress towards threat and safe cues, and expectations of how likely each cue would appear.

### Procedure

After providing informed consent, participants were fitted with SCR and shock electrodes. The level of shock was then adjusted for each participant, starting at 0.3 mA and increasing the current by increments of 0.1 mA (mean current achieved  =  2.6 mA), to arrive at a level of shock that felt “very uncomfortable, but not painful.” Following this, participants were introduced to the experimental task using stimuli that were not repeated during the experimental task. They were told that when a threat cue appeared, they “may or may not” receive an electric shock, and when a safety cue appeared they would never receive a shock. Participants were informed via instructions on the computer screen that they would be subsequently “tested on your [their] ability to remember the personality of these faces, and whether or not they were TRUE or FALSE.” Participants were also told that they would rate their confidence in their response. Participants then received verbal clarification of the learning and memory tasks, and the experimenter clarified what the “true”, “false” options corresponded to for the memory task, and that they would also see some completely new pairings, to which they should respond “never seen”.

Following this, participants completed four experimental blocks with the option of a short break between each block. Each block comprised a single learning and a single memory phase. The interleaving of learning and memory phases was implemented in order not to overly tax memory by testing all items at the end. Upon completion of the experimental blocks, the self-report questions were administered and participants were debriefed.

### Data coding and analysis

Raw data were collected using MATLAB with Psychophysics Toolbox extensions (Brainard 1997; Pelli 1997) and LabChart 8 software (ADInstruments) and were exported into SPSS (IBM Corp, 2017) and R (R Core Team, 2017) for analysis. Twenty-three participants were excluded from analyses due to: equipment failure (n  =  14), failing to meet the threshold for memory performance (average memory performance lower than 2SD’s below the mean, averaged across all trials; n  =  8), and/or SCR non-responders (those who displayed no detectable SCR activity on more than four consecutive threat or shock trials; n  =  3). Two participants met multiple exclusion criteria. A total of 98 participants (*M*_*age*_  =  20.11 years, *SD*  =  4.25) were included in the analyses.

#### Behavioural data

Three dependent variables were calculated. The first was the proportion of non-foil trials participants answered correctly (i.e., true as true, or false or false). In addition, two types of error rates were calculated as a proportion of all non-foil trials: proportion of true/false reversals (i.e., true as false, or false as true) and proportion of trials in which participants said they had “never seen” a pairing that had actually appeared in the learning phase.

#### Physiological response data

Physiological response was indexed by measuring SCR towards threat and safe cues. At each trial of the learning phase, the participant’s SCR was calculated by taking the difference between their averaged SCR in the 2 s before threat or safety cue presentation and their maximum SCR score within the window of 0.3 s—10.3 s post-cue presentation (Braithwaite et al. 2013). This post-cue window was chosen because SCR emerges within approximately 0.3 s, and we allowed a window of up to 10 s for each participant to reach their peak physiological response. The participant’s SCR score was averaged across all threat, safe, and shock trials to determine the final SCR score linked with each trial type. We then applied a square-root transformation to these scores to normalize them for analysis.

## Results

### Memory accuracy

In separate repeated-measures ANOVAs for the three dependent measures (proportion correct, proportion of true/false reversals, and proportion of trials incorrectly labelled as “never seen”), we examined the impact of threat of shock (threat vs. safe), veracity (true vs. false), and valence (positive vs. neutral vs. negative) as within-subjects factors on memory accuracy.

#### Proportion correct

**Fig. 3:**
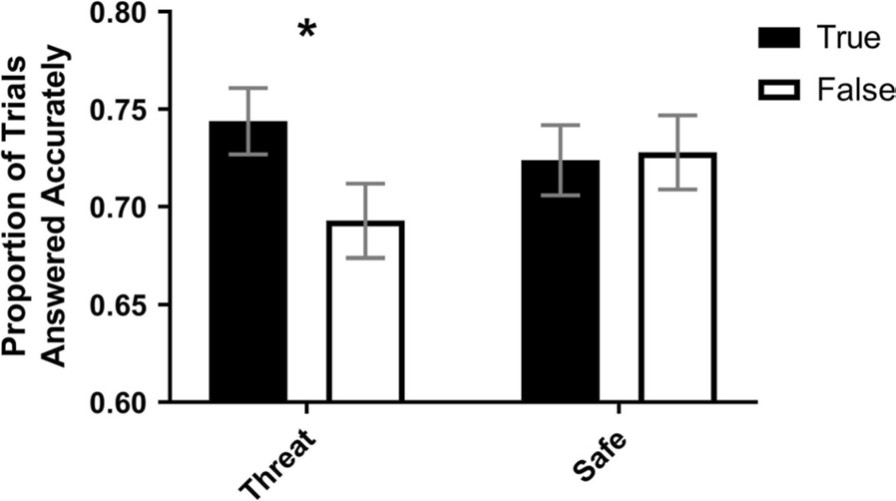
The proportion of memory trials answered correctly by threat of shock and veracity. Error bars represent the standard error

As hypothesized, analysis of the proportion of correct responses revealed a significant veracity x threat of shock interaction, *F*(1,97)  =  4.86, *p*  =  0.030, η_p_^2^  =  0.048. Follow-up pairwise comparisons revealed that response accuracy was significantly lower for trials in which participants had been informed that a pairing was false while under threat (M  =  0.69, SE  =  0.02) compared to trials in which participants had been told that a pairing was true while under threat (M  =  0.74, SE  =  0.02), mean difference (MD)  =  0.051, 95% CI [0.009, 0.093], *p*  =  0.017, but that for safe trials the proportion of correct responses for negated (i.e., “false”) trials (M  =  0.73, SE  =  0.02) did not significantly differ from the proportion of correct responses for non-negated (i.e., “true”) trials (M  =  0.72, SE  =  0.02), MD  =  0.003, 95% CI [−0.036, 0.430], *p*  =  0.866 (see Fig. 3).

A significant veracity x valence interaction also emerged,^1^*F*(1.88,181.94)  =  3.68, *p*  =  0.030, η_p_^2^  =  0.037. Deconstructing this interaction, we found that for positively valenced information, the proportion of correct responses was significantly lower for trials that were negated (M  =  0.68, SE  =  0.02) compared to non-negated (“true”) trials (M  =  0.74, SE  =  0.02), MD  =  −0.05, 95% CI [0.005, 0.100], p  =  0.032. However, for neutral trials we did not see a significant decrease in correct responses for negated trials (M  =  0.71, SE  =  0.02) compared to non-negated trials (M  =  0.76, SE  =  0.02), MD  =  −0.05, 95% CI [0.001, −1.04], p  =  0.057, nor did this pattern emerge for negatively valenced trials that were negated (M  =  0.74, SE  =  0.02) compared to non-negated (M  =  0.70, SE  =  0.02), MD  =  0.03, 95% CI [−0.088, 0.024], *p  *=  0.026. No further main effects or interactions reached significance, including those related to valence (all *p*’s > 0.13).

#### Proportion true/false reversal errors

**Fig. 4:**
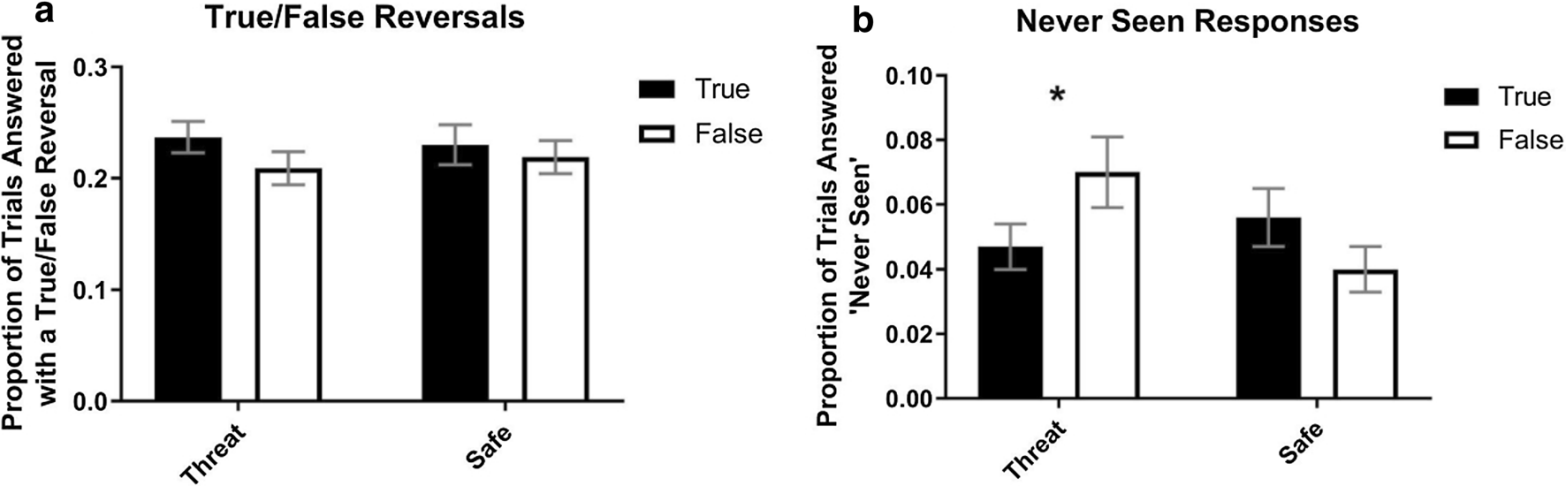
Errors as a function of veracity (true vs. false) and threat of shock (threat vs. safe). Panel A depicts the proportion of errors reflecting true/false reversals (no interaction). Panel B depicts errors whereby participants indicated that they had “never seen” pairings that had actually appeared in the learning phase (two-way interaction and significantly worse memory for FALSE than for TRUE items that had been presented under threat of shock). Error bars represent the standard error

Analysis of the proportion of true/false reversal errors (that is, when participants mistook false items for true items, or true items for false items) revealed no significant veracity x threat of shock interaction, F(1,97)  =  0.49, *p*  =  0.482, η_p_^2^  =  0.005 (see Fig. 4, panel A). This was contrary to our hypotheses that threat of shock would specifically lead participants to mistake false pairings for true ones.

A significant veracity x valence interaction was detected, *F*(2,194)  =  3.21, *p*  =  0.042, η_p_^2^  =  0.032. Echoing the above findings for correct responses, follow-up pairwise comparisons indicated that for positively valenced information, the proportion of true/false reversal responses was significantly higher for trials that were negated (M  =  0.25, SE  =  0.02) compared to non-negated (“true”) trials (M  =  0.21, SE  =  0.02), MD  =  0.05, 95% CI [0.002, 0.090], *p*  =  0.042. This was not the case for neutral or negatively valenced trials. We found no main effect of threat of shock for true/false reversals, *F*(1,97)  =  0.019, *p*  =  0.892, η_p_^2^  =  0.00, nor did any further main effects or interactions reach significance (all *p*’s > 0.19).

#### Proportion of “never seen” errors

However, when examining the proportion of trials in which participants incorrectly answered that they had never seen the pairing before, we observed a significant veracity x threat of shock interaction, *F*(1,97)  =  7.09, *p*  =  0.009, η_p_^2^  =  0.068. Follow-up pairwise comparisons indicated that for threat trials, the proportion of incorrect “never seen” responses was significantly higher for negated trials (i.e., “FALSE”; M  =  0.070, SE  =  0.01) compared to non-negated trials (i.e., “TRUE”; M  =  0.056, SE  =  0.007), MD  =  0.023, 95% CI [0.001, 0.045], *p*  =  0.037. Meanwhile, this was not the case for safe trials, where the proportion of incorrect “never seen” responses did not differ significantly between negated trials (i.e., “FALSE”; M  =  0.042, SE  =  0.007) and non-negated trials (i.e., “TRUE”; M  =  0.056, SE  =  0.009), MD  =  0.014, 95% CI [−0.03, 0.032], *p*  =  0.10 (see Fig. 4, panel B). No further main effects or interactions reached significance (all *p*’s > 0.17).

#### Manipulation checks

We performed a manipulation check on participant’s skin conductance responses (SCR). To determine the impact of threat of shock on participants’ physiological responding, we conducted an ANOVA with threat of shock (safe versus threat of shock) as a within-subjects factor. We observed a significant main effect of threat of shock, *F*(1,97)  =  189.23, *p* < 0.001, η_p_^2^  =  0.66, whereby participants exhibited significantly more physiological arousal during threat of shock trials (M  =  1.13, SE  =  0.05) compared to safe trials (M  =  0.79, SE  =  0.04), MD  =  0.34, 95% CI [0.29, 0.39], *p* < 0.001. SCR responses on shock trials were uninterpretable due to movement artefacts.

#### Self-report measures

Paired samples t tests revealed that participants self-reported significantly more stress when they saw the cue signalling threat of shock (M  =  3.86, SD  =  0.81) compared to the cue signalling safety (M  =  1.50, SD  =  0.80); *t*(97)  =  26.23*, p* < 0.001, d  =  2.65. Participants also reported that their expectations of receiving a shock were significantly greater when the cue signalling threat of shock was presented (M  =  3.50, SD  =  0.97) than when the cue signalling safety was presented (M  =  1.54, SD  =  1.00); *t*(97)  =  15.11*, p* < 0.001, d  =  1.52.

### Confidence ratings

A paired samples t test on mean confidence ratings for correct versus incorrect responses revealed that participants rated their confidence as significantly higher for correct responses (M = 3.82, SD = 0.65) than for incorrect responses (M = 2.81, SD = 0.73); t(97) = 16.90, *p *<. 001, d = 1.72.

Additionally, participants’ confidence ratings for (a) correct and (b) incorrect responses were submitted to two separate 2 (threat of shock: threat vs. safe) × 2 (veracity: true vs. false) × 3 (valence: positive vs. neutral vs. negative) within-subjects ANOVAs.

For correct responses, a significant veracity x threat of shock interaction emerged, *F*(1,78)  =  4.79, *p*  =  0.032, η_p_^2^  =  0.058.^2^ Follow-up pairwise comparisons revealed that for face-descriptor pairings learned under threat of shock, participants had significantly lower confidence for correct responses to non-negated (“true”) pairings (M  =  3.79, SE  =  0.09) than for correct responses to negated pairings (M  =  3.94, SE  =  0.08), MD  =  -−0.15, 95% CI [−0.028, −0.272], *p*  =  0.016). Although this stands in apparent contrast with the patterns of accuracy, it is important to note the small magnitude of the effects involving confidence ratings, which at their largest amounted to 0.17 on a 1–5 rating scale. Given that confidence ratings were not a main outcome of interest in the current study, future work should directly target the interacting effect of arousal and negation of information on confidence for what people remember. Notably, in the absence of threat of shock, participants’ confidence in their responses to non-negated (M  =  3.92, SE  =  0.08) and negated (M  =  3.87, SE  =  0.09) face-descriptor pairings did not significantly differ (MD  =  0.05, 95% CI [−0.094, 0.202], *p*  =  0.47). A significant main effect of valence also emerged, *F*(2,156)  =  7.32, *p*  =  0.001, η_p_^2^  =  0.086. Follow-up pairwise comparisons revealed that participants expressed higher confidence in their responses for neutral descriptors (M  =  3.99, SE  =  0.08) than for positive descriptors (M  =  3.98, SE  =  0.08), MD  =  0.15, 95% CI [0.057, 0.246], *p*  =  0.002, or negative descriptors (M  =  3.81, SE  =  0.08), MD  =  0.17, 95% CI [0.072, 0.271], *p*  =  0.001. Confidence ratings between positive and negative face-descriptors did not significantly differ, *p*  =  0.69. No further main or interaction effects reached significance (all *p*’s > 0.18).

For incorrect responses, no main or interaction effects reached significance (all *p'*s > 0.17).

### Interim summary

Our analysis of the behavioural findings suggests that for memories encoded in safe conditions, participants can update, or correct, representations of the veracity of information with little impact on either negated or non-negated items. Conversely, memory specifically suffers for items that are subsequently negated under looming threat (i.e., threat of shock).

Further research is necessary to draw conclusions about the mechanism underlying this pattern. In contrast to earlier work (e.g., Gilbert et al. 1990), incorrect responses did not necessarily stem from participants miscategorizing negated items as non-negated. Instead, in the present study, these errors seemed to reflect a small number of instances where participants reported never having seen a pairing at all when it had been negated under threat.

### Linear ballistic accumulator modelling

These results suggest that looming threat affects negated representations differently from non-negated memories, but without further analysis it is unclear whether this pattern was due to participants actually having poorer memory of the negated face-descriptor pairings or to decisional processes. For example, it could be that when memories are encoded under threat, participants report these memories more impulsively, leading to more errors when information has been negated.

To help determine what underlay the higher error rate for information negated under threat, we fit our choice and response time data from the memory phase to a linear ballistic accumulator (LBA) race model (Brown and Heathcote 2008). To keep the model tractable, and as word valence was not directly relevant to assessing this determination, we did not include any effect of word valence in the LBA model (though trials from all valences were included during model fitting). The LBA assumes that when making a response from a selection of alternatives, each alternative is represented by an *accumulator*. These accumulators race against each other, with the speed of each accumulator given by its *drift rate*. Drift rate is determined by the amount of evidence in favour of each alternative: the more evidence for one alternative over another (in this case, the stronger the memory), the higher its drift rate will be. At some point in the race, one of the accumulators reaches a predetermined *threshold*, whereupon it "wins" the race and is selected. The higher the threshold for an alternative is, the more evidence that must be collected for that alternative to win the race, and as more evidence is required, the less such decisions are susceptible to decision noise, or "gut feeling"/flippant responses. Conversely, lower thresholds lead to more decision noise and fewer correct responses.

**Fig. 5:**
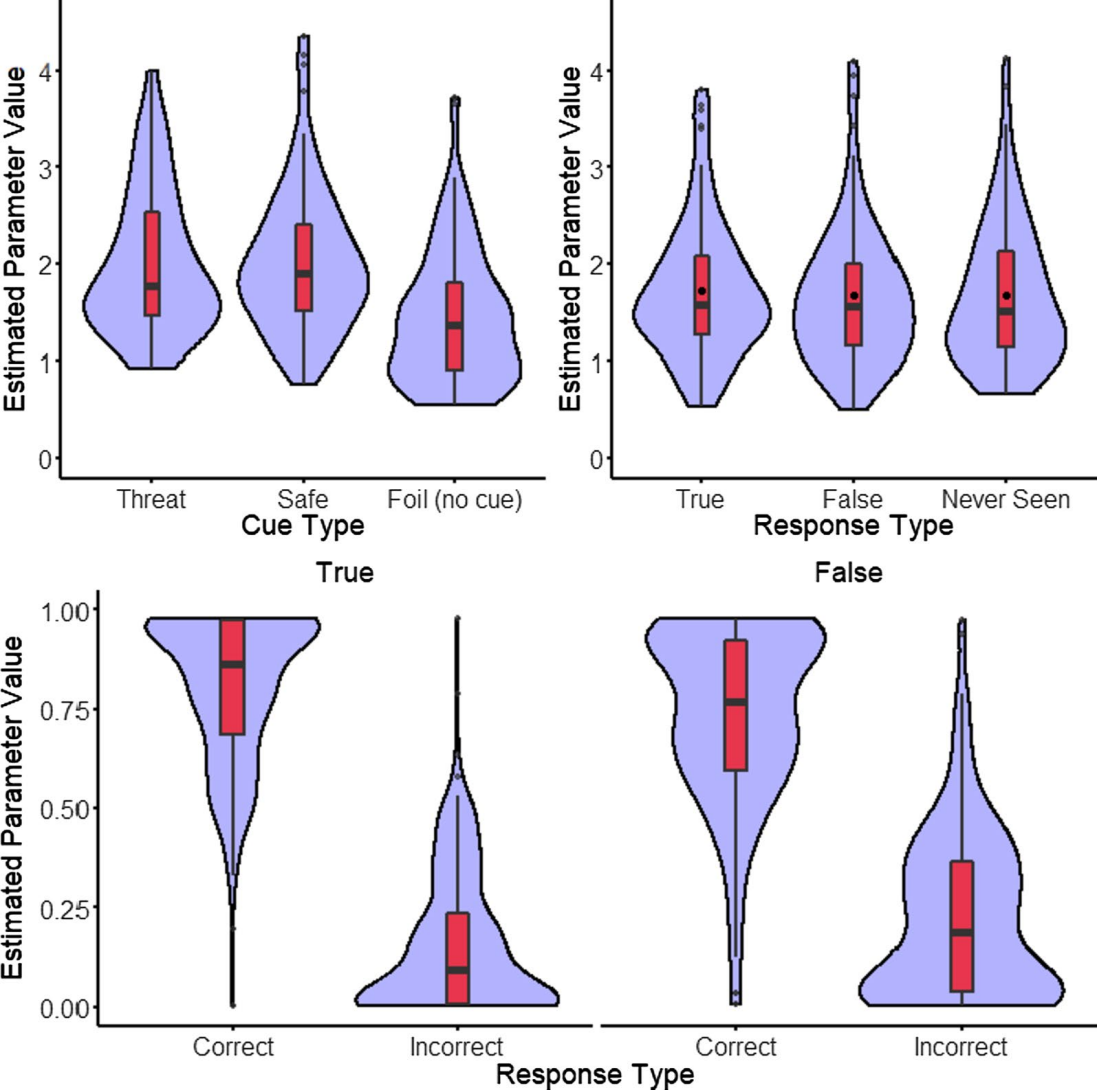
**Panel a** aggregated parameter estimates for thresholds of different cue types; **Panel** **b** aggregated parameter estimates for thresholds of the different possible responses; **Panel c** aggregated parameter estimates for drift rates for correct/incorrect responses in non-negated (true) trials and negated (false) trials. Box plot hinges extend from the 1st to the 3rd quartile; whiskers extend up to 1.5 times the interquartile range. The blue region shows the density of the values that were estimated for each parameter across participants

The aggregated parameter estimates for the thresholds (Fig. 5a) suggested no difference between thresholds for threat trials versus safe trials, and this was supported by our analyses, *t*(97) = 0*.*26*, p* = *.*794. (The full report of all modelling methods and analyses can be found in the Additional file 1). We also found no difference in the thresholds for any of the different response types (correct versus true/false reversal versus never seen; Fig. 5b), *F*(2*,*194) = 2*.*35*, p* = 0*.*098. This indicated that participants did not differ in their level of impulsiveness when responding to threat or safe trials and that participants were not biased to make any particular response type. However, as suggested by Fig. 5a we found that participants required less evidence on foil trials (where the face-descriptor pairing had not been previously seen) before making a response compared to threat trials, *t*(97) = 14*.*63*, p *<*. *001, or safe trials, *t*(97) = 14*.*31*, p *<*. *001, indicating that participants appeared to be able to recognize when they had not seen a face-descriptor pairing before, and required less evidence when making a response on those trials.

In drift rate, there was a clear effect of response type (correct versus true/false reversal versus never seen responses), *F*(2*,*194)  =  743*.*43*, p* < 0.001, with faster drift rates for correct responses than incorrect responses (true/false reversals, *t*(391)  =  28.77, *p* < 0.001, and faster drift rates for incorrect trials than never-seen trials, *t*(391)  =  14.25, *p* < 0.001. We also observed a response type x veracity interaction (Fig. 5c), *F*(2*,*194)  =  10*.*67*, p* < 0.001, with negated trials having lower drift rates for the correct response and higher drift rates for the incorrect response compared to non-negated trials. This indicated that participants overall had a poorer memory for negated (“false”) trials than for non-negated (“true”) trials, in line with the behavioural findings. We did not observe any veracity x threat of shock interaction, threat of shock x response type interaction, or three-way interaction (all *p*’s > 0.48). This suggests that although memory for negated items was overall worse than memory for non-negated items, there was no evidence that memory was affected by threat of shock in the model.

## Discussion

The current study investigated the impact of looming threat on people’s ability to recategorize information as untrue once it had been negated after an initial presentation. On each trial, participants saw a face paired with a descriptor and each pairing was followed by a verification of the descriptor or a negation, indicating that the descriptor was false. Crucially, to assess whether the cognitive process of applying a negation is weakened by looming threat, this verification/negation was sometimes immediately followed by a cue indicating the possibility of receiving an imminent electric shock.

When subsequently tested on their memory for the face-descriptor pairs, selective impairments emerged. That is, the looming threat of shock specifically impaired participants’ memory for face-descriptor pairings that had been followed by a negation. In contrast, the threat of shock did not impair their ability to remember pairings that were not negated.

At first glance, these findings appear to confirm our predictions, as we had hypothesized that participants would mistake false pairings for true ones (Gilbert et al. 1990). However, closer examination revealed that although participants were indeed worse at remembering pairings that had been negated under looming threat, this could not be attributed to participants mistaking negated pairings for true pairings (i.e., failing to apply the negation). Instead, a small but significant pattern suggested that participants sometimes failed to recognize that they had previously seen the negated pairings at all. This pattern does not rule out the possibility that other mechanisms may contribute to worse memory for items negated under threat (e.g., those identified by Gilbert et al. 1990), but such potential additional mechanisms did not reveal themselves in the current study.

In terms of mechanism, aspects of the current design are reminiscent of a phenomenon known as “directed forgetting”, in which people are instructed to forget a subset of learned material and are subsequently worse at recalling that material on a memory test (e.g., Bjork et al. 1968; MacLeod 2012). In the current study, a negation following a face-descriptor pair could arguably be interpreted as an implicit instruction to forget the pair. However, negative emotions have been found to diminish the directed forgetting effect, possibly due to impaired cognitive processes that would otherwise enable it (Minnema and Knowlton 2008), and this contrasts with the current study, where looming threat *increased* forgetting of negated material.

A potential alternative explanation of the current findings may be found in the literature examining how competing representations mutually suppress one another in memory. In a widely studied task (known as the A-B, A-C learning paradigm), participants learn an association between two items (i.e., A-B), and then learn an association between either two new items (C-D) or one new and one old item (A-C). Findings from this task have revealed that interference arises due to competition between items that are linked to a shared associate—e.g., competition between “B” and “C” when they have both been linked with “A”—and that this competition increases retrieval difficulty (Mensink and Raaijmakers 1988). Executive control systems, involving the prefrontal cortex, play an important role in resolving such competition and aiding retrieval (Badre and Wagner 2007; Kuhl et al. 2008). In the context of the present study, and consistent with evidence that people initially accept a statement as true upon receiving it (Gilbert et al. 1990), being subsequently presented with information that a statement is in fact false may temporarily place people in the position of holding two simultaneous representations of a piece of information, one true and one false. Whereas participants would normally apply executive control to resolve this competition, stress and anxiety have been found to interfere with frontal lobe function, executive control, and the resolution of interference (Arnsten and Goldman-Rakic 1998; Choi et al. 2012; Eysenck et al. 2007; Park and Moghaddam 2017). Thus, cues indicating possibly imminent shock may be stressful or arousing enough to interfere with participants’ ability to resolve the competition between the “true” and “false” tags linked with the information, leading to continued mutual interference between those competing representations—and therefore to more forgetting of them. This account reflects a post hoc interpretation of the current findings; it does not conform to our a priori predictions. Yet, linear ballistic accumulator (LBA) modelling of the data supports this interpretation, suggesting that participants’ diminished ability to remember information that had been negated was due to a weaker memory trace for those items. Future work might be able to more rigorously, in a priori fashion, test whether mutually suppressive representations underlie such forgetting by explicitly introducing a competing representation (e.g., “Jane is friendly”) instead of simply a negation of an initial statement (e.g., that the statement “Jane is mean” is false).

In sum, in the present study, when people were confronted with negations of false information, they were less likely to remember having seen the information at all when the negation co-occurred with looming threat (i.e., threat of shock). In contrast, no such memory impairment occurred for non-negated information or when negations occurred under safe conditions. This observed pattern extends a growing literature on cognitive mechanisms that render it challenging to correct misinformation (Lewandowsky et al. 2012). One open question is whether corrections under looming threat might be more successful when they substitute correct for incorrect information rather than simply negate the incorrect information (e.g., “Jane is actually KIND” rather than “Jane is NOT mean”; see Mayo et al. 2004). Future work might also examine whether the impact of anxious anticipation on processing of negated information diminishes when anxiety is sustained over long periods of time.

To the degree that these findings generalize to the real world, they have important implications. In the 24-h news cycle that characterizes modern society, information comes at such a rapid pace that retractions and corrections are common. This may be especially the case when the information pertains to anxiety- or stress-provoking situations, such as a developing pandemic. In these situations, people rely on accurate, updated information to know how best to take care of themselves and others. It may be particularly important, when important information pertains to anxiety- and stress-provoking situations, for those responsible for communicating advice and guidelines to verify their accuracy beforehand instead of relying on corrective updates.

## Supplementary Information

**Additional file 1**. Supplementary information.

## Data Availability

The complete dataset supporting the conclusions of this article here is available in the OSF repository, available online at https://osf.io/m34n9/.

## Footnotes

1 We applied a Greenhouse–Geisser correction to account for an observed violation of sphericity, χ2  =  6.58, *p*  =  .037.

2 Note that the degrees of freedom are different for this analysis due to missing data points for some participants, who did not provide confidence ratings for specific trial types (as they did not get a “false” pairing with a negative word under threat of shock correct, for example).
