# Supplementary material for: Out of fright, out of mind: impaired memory for information negated during looming threat
Source: Cogn Res Princ Implic. 2021 May 7;6:36. doi: 10.1186/s41235-021-00302-4 (PMC8102851; doi:10.1186/s41235-021-00302-4)
Supplement: Supplementary file 1 — Additional file 1. Supplementary information. [file 41235_2021_302_MOESM1_ESM.docx]

# Supplementary Materials

**Results**

**Participant demographics.** Mean sample characteristics are reported in Table S1.

**Table S1.** Mean participant characteristics

|  | **Sample** |
| --- | --- |
| Final Sample size | *n* = 98 |
| Age | 20.11 (4.25) |
| Depression | 9.83 (3.77) |
| Anxiety | 9.60 (2.97) |
| Stress | 11.43 (3.90) |
| Trait optimism and pessimism | 26.06 (4.12) |

Note: Standard deviations appear in parentheses.

**Impact of shock on memory.** We performed an additional analysis on ‘Shock’ trials, where participants received a shock, to determine the impact of an actual interruption on memory recall. In these trials, an impairment in memory for ‘false’ trials due to shock emerged, in the form of a significant main effect of Veracity, *F*(1,97)=4.75, *p*=.032, η_p_^2^=0.047. This indicates that memory for negated (“false”) pairings was significantly reduced (M=0.69, SE=0.02) compared to recall for non-negated (“true”) pairings (M=0.75, SE=0.02).

## Additional Details - Linear Ballistic Accumulator Model

The Linear Ballistic Accumulator (LBA, Brown & Heathcote, 2008) provided a way to assess whether the difference in participant performance in threat trials is driven by a poorer memory for false corrected items (reflected by lower drift-rates for correct responses), or less diligence when making responses under threat (reflected by lower thresholds for threat trials over safe trials). The model also included a parameter for non-decision time in which no accumulation takes place (t0), A start-point parameter that dictates the range in which accumulation can begin on each trial (A), and a parameter that measures the standard deviation of the drift-rate (s). A more in-depth explanation of the model and its general solution can be found in Brown and Heathcote (2008).

We fit our choice and response time data from the memory phase to the LBA using the rtdists package in R (R Core Team, 2015; Singmann, Brown, Gretton, & Heathcote, 2016) with a differential evolution algorithm from the DEoptim package (Ardia, Arango, & Gomez, 2011; Ardia, Boudt, Carl, Mullen, & Peterson, 2011; Ardia, Mullen, Peterson, & Ulrich, 2016; Mullen, Ardia, Gil, Windover, & Cline, 2011; Price, Storn, & Lampinen, 2006). For each participant, 500 iterations of the genetic algorithm were completed. We estimated a threshold (B) for each participant for each different response (Uncorrected True, Corrected False, Never Seen), as well as each level of threat (Threat, Safe, and Foil (no cue)). We did not include any effect of word valence in our model (though trials from all valences were included during model fitting). Thresholds values during fitting were restricted with a lower bound of .5, and an upper bound of 5 (expressed from here on as [.5, 5]). Thresholds for each trial were determined by adding the threshold for relevant threat level to the threshold for each response.

We estimated a drift rate (d) for each participant for each response under each level of ground truth veracity (Uncorrected True, Corrected False, Never Seen) and each level of threat. To decrease fitting time, we fixed the threshold for the Never Seen responses to 1, and freely estimated the other drift rates [.1, 50]. For fitting and analysis, drift-rates were normalised so that they equaled to 1 for each set of racing accumulators on each trial, given by the formula

$$d_{x}=\frac{d_{x}}{d_{true}+ d_{false}+ d_{ns}}$$

where dX is the drift rate being estimated. Finally, we estimated one t0 [.1, 1], A [0, 10], and s [0, 2] parameter for each participant. This meant that we estimated 23 parameters in total for each participant. To examine the difference between thresholds for both cue type (Main text Figure 5A) and response type (Main text Figure 5B), we ran 2 repeated-measures ANOVAs using the ’ez’ package in R (Lawrence, 2016). We found a significant effect of cue type, F(2,194)=143.03, p<.001, with follow-up Bonferroni corrected pair-wise t-tests showing a significant difference between the thresholds for threat and foil cues, t(97)=14.63, p<.001, and safe cues, t(97)=14.31, p<.001. There was no significant difference observed between thresholds for threat cues and safe cues, t(97)=0.26, p=.794. For the analysis of response type, we found no differences in the thresholds for any of the response types, F(2,194)=2.35, p=.098.

To examine the difference between correct and incorrect response drift-rates (Main text Figure 5C), we re-organised our estimated drift-rates, such that the ground-truth veracity on that trial was now coded as the "correct" response, and the alternative response was coded as "incorrect". Notably, we did not include estimates of Never Seen responses in the calculation of "incorrect" responses, as we were theoretically interested in the alteration of the memory for previously seen items. Instead, they were treated as a separate category in the analysis. For the same reason, we did not include estimates of drift-rates for foil trials as a category in our analysis.

We ran a 2 (Threat of Shock) x 2 (Veracity) x 3 (Response Type) repeated measures ANOVA. As it was a requirement that the sum of all drift-rates equalled one for a given trial type, the main-effect of cue-type and veracity were non-interpretable (as the sum of drift rates for all safe and threat trials equalled one, and similarly for the uncorrected true/corrected false trials). We observed a main effect of response type, F(2,194)=743.43, p<.001, with drift rates higher for correct than incorrect trials, t(391)=28.77, p<.001, and incorrect trials higher than never-seen trials, t(391)=14.25, p<.001. For the interactions, only the veracity by response type interaction was significant, F(2,194)=10.67, p<.001 (all other interactions ps<.477). Follow-up t-tests with Bonferroni corrections revealed that drift rates were lower for correct responses when the ground-truth veracity was corrected to false compared to when it was uncorrected true, t(97)=3.13, p=0.007, and conversely higher for incorrect responses when ground-truth veracity was false compared to when it was true, t(97)=2.75, p=0.021. Importantly, we did not observe a difference in drift-rates between veracities for never seen responses, t(97)=1.22, p=0.680, indicating that the decrease in performance for false trials was driven by participants appearing to remember the item had been in the list they had seen, but more often remembering the incorrect veracity compared to true trials.

## Stimulus Selection

**Face stimuli selection.** Face stimuli were obtained from the Glasgow Unfamiliar Face Database (Burton, White, McNeill, 2010). This involved obtaining pairwise similarity data from a previous pile sorting task conducted by Burton and his colleagues (2010). This data were subject to a multidimensional scaling analysis, the output of which was then visually represented via a scatterplot according to the sex of the face image. From the female plot, we selected 84 faces with the largest spatial distance (least visual similarity) amongst each other. To optimise participants’ ability to distinguish between faces, we equally divided our set of 84 face images into two sets using the same selection methods described above. Each set was used across two consecutive blocks, where a face from the assigned set was randomly assigned to a descriptor at each trial. Allocation of face sets to the first two or last two blocks were counterbalanced across participants.

**Word stimuli selection.** Face-descriptors were selected from a list of 13,915 English words rated on measures of valence and arousal (Warriner et al., 2013). Various steps were undertaken as follows. First, Warriner and colleague’s (2013) list was re-sorted according to descending valence. Then, we extracted 200 adjectives from the top of the list (positive words), 300 adjectives from the middle of the list (neutral words), and 200 adjectives from the bottom of the list (negative words). Words that did not contain concreteness or frequency ratings from the respective databases (outlined below) were then excluded from the lists. Then, each list was sorted according to descending word length, where the middle third of each list was extracted. These lists of words were sorted according to descending frequency, from which the middle third of this list was extracted. Lastly, the middle 28 words from each list was extracted to yield the final positive, neutral and negative list of face-descriptors. Valence and arousal ratings were obtained from Warriner and colleagues’ (2013) list, frequency ratings from the CELEX2 database (Baayen, Piepenbrock, & Guilikers, 1996) and concreteness ratings from Brysbaert, Warriner and Kuperman (2014).

Mean valence ratings for positive words were significantly higher compared to neutral, followed by negative words. Further, the mean arousal ratings of positive and negative words were equated and were higher than those of neutral words. All words were controlled for frequency, concreteness and word length. See Table S2 for average word ratings, Table S3 for statistical comparisons between stimulus types, and Table S4 for all descriptor words.

**Table S2.** Average word length and ratings of arousal, frequency and concreteness for positive, neutral and negative emotional words

| Word type | Valence | Arousal | Frequency | Concreteness | Word Length |
| --- | --- | --- | --- | --- | --- |
| Positive | 7.49 | 4.74 | 11.61 | 2.08 | 7.71 |
| Neutral | 5.14 | 4.15 | 7.74 | 2.27 | 7.39 |
| Negative | 2.41 | 4.93 | 9.70 | 2.10 | 7.79 |

**Table S3.** Two-tailed *t*-test comparing ratings of word valence, arousal, frequency, concreteness, and word length of word stimuli

|  |  | Sig (*p-*value) |
| --- | --- | --- |
| Valence | Pos vs Neu | <.001** |
|  | Pos vs Neg | <.001** |
|  | Neu vs Neg | <.001** |
| Arousal | Pos vs Neu | .034* |
|  | Pos vs Neg | .461 |
|  | Neu vs Neg | .003** |
| Frequency | Pos vs Neu | .075 |
|  | Pos vs Neg | .414 |
|  | Neu vs Neg | .362 |
| Concreteness | Pos vs Neu | .052 |
|  | Pos vs Neg | .822 |
|  | Neu vs Neg | .074 |
| Word length | Pos vs Neu | .474 |
|  | Pos vs Neg | .867 |
|  | Neu vs Neg | .413 |

** *p* < .001 ** *p* < .01 * *p* < .05

**Table S4.** Word length and ratings of arousal, frequency and concreteness for positive, neutral and negative emotional words

| Positive word | Valence | Arousal | Frequency | Concreteness | Word Length |
| --- | --- | --- | --- | --- | --- |
| Helpful | 7.33 | 3.61 | 26.31 | 1.76 | 7 |
| Polite | 6.4 | 3.45 | 21.34 | 1.85 | 6 |
| Joyful | 8.24 | 5.72 | 3.58 | 2.12 | 6 |
| Faithful | 8.23 | 3.83 | 13.74 | 2.2 | 8 |
| Charming | 8.22 | 5.54 | 22.57 | 2.14 | 8 |
| Cheerful | 8.1 | 6.14 | 18.49 | 2.34 | 8 |
| Peaceful | 8.06 | 4.82 | 20.45 | 2.27 | 8 |
| Vibrant | 8 | 6.13 | 2.4 | 2.38 | 7 |
| Admirable | 7.25 | 4.18 | 10.61 | 1.81 | 9 |
| Playful | 8 | 5.5 | 2.96 | 2.63 | 7 |
| Optimistic | 7.08 | 4.56 | 10.67 | 2 | 10 |
| Delightful | 7.62 | 6 | 13.07 | 2.38 | 10 |
| Courteous | 6.7 | 3.62 | 5.25 | 1.97 | 9 |
| Witty | 7 | 5.64 | 6.03 | 2.21 | 5 |
| Authentic | 7.23 | 4.21 | 8.04 | 2.31 | 9 |
| Lively | 7.16 | 5.2 | 13.74 | 2.36 | 6 |
| Noble | 7.3 | 3.71 | 17.21 | 1.89 | 5 |
| Humorous | 8.22 | 5.07 | 5.31 | 2.23 | 8 |
| Selfless | 7.3 | 4.33 | 1.68 | 1.61 | 8 |
| Thoughtful | 7.61 | 2.37 | 10.11 | 1.96 | 10 |
| Carefree | 7.4 | 4.08 | 1.62 | 1.88 | 8 |
| Endearing | 7.6 | 3.73 | 1.9 | 1.9 | 9 |
| Sincere | 6.77 | 3.64 | 8.49 | 1.81 | 7 |
| Generous | 7.4 | 5.58 | 25.53 | 2.25 | 8 |
| Clever | 7.22 | 5 | 31.73 | 1.79 | 6 |
| Radiant | 7.35 | 4.79 | 4.92 | 2.45 | 7 |
| Passionate | 7.11 | 6.46 | 15.59 | 1.48 | 10 |
| Adoring | 7.83 | 5.71 | 1.62 | 2.34 | 7 |

| Neutral word | Valence | Arousal | Frequency | Concreteness | Word Length |
| --- | --- | --- | --- | --- | --- |
| Expressive | 5.57 | 5.07 | 6.37 | 2.03 | 10 |
| Grandiose | 4.92 | 4.75 | 2.51 | 2.38 | 9 |
| Meticulous | 5 | 5 | 4.47 | 1.9 | 10 |
| Drowsy | 4.5 | 2.36 | 2.85 | 2.62 | 6 |
| Prudent | 5.33 | 3.17 | 7.09 | 1.77 | 7 |
| Alert | 5.58 | 4.9 | 18.16 | 2.59 | 5 |
| Fragile | 5.18 | 3.33 | 11.56 | 2.86 | 7 |
| Swift | 5.52 | 4.78 | 14.41 | 3.04 | 5 |
| Agile | 5.5 | 4.71 | 2.12 | 2.31 | 5 |
| Underrated | 5.3 | 4.67 | 0.61 | 1.93 | 10 |
| Sporty | 5.08 | 4.67 | 0.61 | 2.62 | 6 |
| Flashy | 4.82 | 6 | 1.45 | 2.86 | 6 |
| Chatty | 4.5 | 4.93 | 1.17 | 2.76 | 6 |
| Sleepy | 4.77 | 2.5 | 7.93 | 2.77 | 6 |
| Flamboyant | 5 | 6.14 | 3.46 | 2.18 | 10 |
| Insistent | 4.85 | 3.17 | 4.36 | 1.72 | 9 |
| Unmarried | 5 | 4 | 7.54 | 2.82 | 9 |
| Reserved | 5.75 | 3.08 | 16.03 | 2.04 | 8 |
| Frivolous | 5.07 | 4.58 | 5.03 | 2.43 | 9 |
| Exclusive | 4.82 | 5.25 | 13.52 | 2.11 | 9 |
| Candid | 5.5 | 3.87 | 2.29 | 1.81 | 6 |
| Bashful | 5.5 | 3.83 | 0.78 | 2.19 | 7 |
| Reverent | 5.12 | 4 | 1.17 | 1.5 | 8 |
| Rational | 4.67 | 3.5 | 27.82 | 1.62 | 8 |
| Shy | 5.4 | 3.38 | 18.04 | 2.52 | 3 |
| Lenient | 5.5 | 3.33 | 1.62 | 1.96 | 7 |
| Passive | 4.5 | 3.21 | 16.26 | 2.26 | 7 |
| Realistic | 5.7 | 4.14 | 17.37 | 2.07 | 9 |

| Negative word | Valence | Arousal | Frequency | Concreteness | Word Length |
| --- | --- | --- | --- | --- | --- |
| Creepy | 2.5 | 5.69 | 0.89 | 2.33 | 6 |
| Annoying | 3.15 | 5.78 | 5.36 | 2.31 | 8 |
| Unbearable | 2.22 | 5.5 | 6.03 | 2 | 10 |
| Sickening | 2.38 | 5.43 | 3.07 | 2.32 | 9 |
| Stingy | 2.62 | 4.64 | 0.56 | 2 | 6 |
| Wretched | 1.92 | 3.78 | 12.35 | 2.03 | 8 |
| Rude | 1.81 | 5.6 | 12.68 | 2.52 | 4 |
| Deceitful | 2.15 | 5.58 | 1.28 | 2.13 | 9 |
| Ignorant | 2.64 | 4.6 | 15.47 | 1.62 | 8 |
| Shameful | 2.86 | 4.91 | 5.36 | 1.71 | 8 |
| Unfriendly | 2.5 | 4.25 | 2.96 | 2.12 | 10 |
| Depressing | 2.4 | 2.64 | 9.27 | 1.79 | 10 |
| Dreadful | 3 | 4.17 | 22.74 | 2.23 | 8 |
| Nasty | 3.44 | 5.73 | 23.46 | 2.37 | 5 |
| Foolish | 2.69 | 4.67 | 20.06 | 1.84 | 7 |
| Greedy | 1.58 | 5.42 | 7.04 | 2.64 | 6 |
| Abusive | 2.09 | 5.56 | 1.51 | 1.84 | 7 |
| Malicious | 2.12 | 5.08 | 5.03 | 2.33 | 9 |
| Unreliable | 2.75 | 3.69 | 4.36 | 1.83 | 10 |
| Devious | 3.31 | 3.29 | 3.07 | 2.18 | 7 |
| Disgusting | 1.67 | 6.31 | 9.89 | 2.21 | 10 |
| Unpleasant | 2.23 | 4.27 | 21.01 | 1.91 | 10 |
| Cruel | 2.43 | 5.62 | 27.04 | 2.11 | 5 |
| Negligent | 1.8 | 4.33 | 0.61 | 1.92 | 9 |
| Jealous | 2.12 | 5.92 | 18.6 | 2.17 | 7 |
| Disloyal | 2.33 | 4.86 | 1.56 | 1.64 | 8 |
| Hateful | 2 | 5.62 | 3.74 | 2.07 | 7 |
| Hostile | 2.67 | 5 | 26.59 | 2.62 | 7 |

#

# Supplementary References

Ardia, D., Arango, J. O., & Gomez, N. G. (2011). {J}ump-Diffusion Calibration using{D}ifferential {E}volution.Wilmott Magazine,55, 76–79.

Ardia, D., Boudt, K., Carl, P., Mullen, K. M., & Peterson, B. G. (2011). {D}ifferential{E}volution with {DEoptim}: An Application to Non-Convex Portfolio Optimization. The R Journal,3(1), 27–34.

Ardia, D., Mullen, K. M., Peterson, B. G., & Ulrich, J. (2016). {DEoptim}: Differential Evolution in {R} [Computer software manual]. Retrieved fromhttps://cran.r-project.org/package=DEoptim

Baayen, R.H., Piepenbrock, R., and Guilikers, L. (1996). CELEX2 Database (CD-ROM). Linguistic Data Consortium, http://www.ldc.upenn.edu/.

Brown, S.D., & Heathcote, A. (2008). The simplest complete model of choice response time: linear ballistic accumulation. Cognitive Psychology, 57(3), 153-178. doi: 10.1016/j.cogpsych.2007.12.002

Brysbaert, M., Warriner, A. B., & Kuperman, V. (2014). Concreteness ratings for 40 thousand generally known English word lemmas. *Behavior research methods*, *46*(3), 904–911. https://doi.org/10.3758/s13428-013-0403-5

Burton, A., White, D., & McNeill, A. (2010). The Glasgow Face Matching Test. Behavior Research Methods, 42(1), 286-291. http://dx.doi.org/10.3758/brm.42.1.286

Lawrence, M. A. (2016). ez: Easy Analysis and Visualization of Factorial Experiments [Computer software manual]. Retrieved from <https://cran.r-project.org/package=ez>.

Mullen, K., Ardia, D., Gil, D., Windover, D., & Cline, J. (2011). {DEoptim}: An {R}Package for Global Optimization by Differential Evolution. Journal of Statistical Software, 40(6), 1–26.

Price, K. V., Storn, R. M., & Lampinen, J. A. (2006). {D}ifferential {E}volution - A Practical Approach to Global Optimization. Springer-Verlag.

R Core Team (2015). R: A language and environment for statistical computing. R Foundation for Statistical Computing, Vienna, Austria.

Singmann, H., Brown, S., Gretton, M., & Heathcote, A. (2016). rtdists: Response Time Distributions [Computer software manual]. Retrieved from <https://cran.r-project.org/package=rtdists>

Warriner, A., Kuperman, V., & Brysbaert, M. (2013). Norms of valence, arousal, and dominance for 13,915 English lemmas. *Behavior Research Methods*, *45*(4), 1191-1207. http://dx.doi.org/10.3758/s13428-012-0314-x
